# Positive psychological changes and associated factors among community health workers post-COVID-19 in China: a mixed-methods study

**DOI:** 10.3389/fpubh.2026.1918732

**Published:** 2026-08-28

**Authors:** Qingyu Li, Yuhang Zhang, Jiaruo Sun, Jingtao Zhou, Siwen Huang, Liqun Wu, Wannian Liang, Jiming Zhu, Huatang Zeng, Sitong Luo

**Affiliations:** 1 Vanke School of Public Health, Tsinghua University, Beijing, China; 2 Health Commission of Dalian, Dalian, Liaoning, China; 3 Shenzhen Health Development Research and Data Management Center, Shenzhen, Guangdong, China; 4 General Practice School, Southern University of Science and Technology, Shenzhen, Guangdong, China; 5 Institute for Healthy China, Tsinghua University, Beijing, China

**Keywords:** community health workers, coping strategies, COVID-19, deliberate rumination, mixed-methods study, positive psychological changes, primary health care, social support

## Abstract

**Background:**

Community health workers (CHWs) played an important role in community-based COVID-19 prevention and control while maintaining routine primary care. The pandemic created a stressful work context for CHWs and may also have been followed by perceived positive psychological changes. Although positive psychological changes have been studied among hospital-based healthcare workers, less is known about such changes among CHWs in China. This study assessed self-reported positive psychological changes and associated factors among Chinese CHWs after the COVID-19 pandemic.

**Methods:**

A mixed-methods study was conducted from April 29 to June 10, 2023, in Shenzhen, China. In the quantitative phase, cluster sampling was used to recruit CHWs from 51 community health centers. Participants completed an online survey on demographic characteristics, COVID-19-related exposure, rumination, coping strategies, perceived social support, and positive psychological changes. Moderate-to-high positive psychological changes were defined as a mean item score of ≥3 in at least one domain of the Chinese version of the Post-Traumatic Growth Inventory. Univariate and multivariable logistic regression analyses were performed. In the qualitative phase, purposive sampling was used to recruit CHWs for semi-structured interviews, and interview data were analyzed using interpretative phenomenological analysis.

**Results:**

The quantitative phase included 634 CHWs. The mean age was 35.7 years, and 76.0% were female. Overall, 58.8% reported moderate-to-high positive psychological change. After adjustment, moderate-to-high positive psychological change was associated with higher positive coping (AOR = 1.13, 95% CI: 1.08–1.18), deliberate rumination (AOR = 1.12, 95% CI: 1.06–1.18), and family support (AOR = 1.08, 95% CI: 1.02–1.14). Sensitivity analysis showed similar patterns, although some associations differed between the models. The qualitative phase included 12 CHWs from 6 community health centers. Interviews further suggested that coping strategies, cognitive processing, family support, and work-related support shaped their psychological adjustment.

**Conclusion:**

More than half of CHWs reported moderate-to-high positive psychological change after the COVID-19 pandemic. Workplace mental health programs for CHWs should address distress while supporting adaptive coping, reflective processing of difficult work experiences, and family-sensitive support. These measures may help protect CHWs’ mental health and improve primary care preparedness for future public health emergencies.

## 1 Background

The coronavirus disease 2019 (COVID-19) pandemic was a major public health crisis that placed long-term pressure on health systems worldwide. In addition to its direct effects on physical health, the pandemic was associated with a wide range of psychological problems, especially among healthcare workers, who faced higher occupational exposure, heavier workloads, and prolonged work-related stress than the general population (1, 2). Although the acute phase of the pandemic has ended, its psychological and occupational effects on healthcare workers may continue into the post-pandemic period. Studying psychological responses after the pandemic remains timely, as this period offers an opportunity to understand how healthcare workers make sense of their pandemic-related experiences and how health systems can support their psychological adjustment and preparedness for future public health emergencies (3–5).

Community health workers (CHWs) played an important role in local pandemic response, particularly in primary care and community-based prevention and control. In China, CHWs undertook both routine primary health care responsibilities and COVID-19-related public health tasks, such as early case detection, nucleic acid testing, vaccination, health education, and community follow-up. Between 2020 and 2023, primary healthcare institutions provided more than half of all medical consultations in China, reflecting the important role of community-based health services during this period. Existing studies reported that about one-third had at least one psychological symptom during the pandemic, suggesting that this workforce group may have been particularly vulnerable to the psychological burden of the pandemic (6). Compared with hospital-based healthcare workers, CHWs were positioned at the interface between the health system and local communities (7, 8). They were required to maintain routine primary care while responding to rapidly changing public health tasks, frequent resident contact, community surveillance, and follow-up responsibilities (9). These work experiences may have shaped not only psychological distress but also perceived positive psychological changes, professional identity, relationships with residents, and views about the meaning of primary care work (10, 11).

The COVID-19 pandemic was a public health emergency and may have constituted a potentially traumatic or highly stressful collective crisis for many healthcare workers (12). It exposed healthcare workers to infection risk, heavy workload, uncertainty, social isolation, and repeated contact with fear, illness, and death (13, 14). For CHWs, these pressures were closely linked to their frontline community-based responsibilities during a rapidly changing public health emergency (7). Therefore, although the pandemic should not be assumed to have been experienced as trauma by all individuals, it provided a crisis context in which traumatic stress and positive psychological change could both occur (3).

Positive psychological changes may be perceived after individuals experience and adapt to traumatic, highly stressful, or life-challenging events (3). The posttraumatic growth framework provides one theoretical perspective for understanding how such changes may arise following adversity. According to the revised posttraumatic growth model, major crises may challenge individuals’ core beliefs and life goals, including assumptions about health, safety, personal control, relationships, and the predictability of everyday life (15, 16). Such disruption may first lead to emotional distress and intrusive thinking about the event (17). Over time, deliberate rumination and meaning reconstruction may help individuals reinterpret the experience and develop positive changes in life priorities, interpersonal relationships, perceived personal strength, future possibilities, and self-understanding (18). Perceived positive psychological changes do not imply the absence of distress, nor do they mean that the event itself is beneficial. Rather, positive and negative psychological responses may coexist as individuals adapt to adversity (17, 18). Meanwhile, adverse experiences may also be followed by perceived negative changes, often described as posttraumatic depreciation (19). Therefore, perceived positive psychological changes should be understood as one possible response rather than as the only psychological outcome (20).

Previous studies have shown that positive psychological changes may occur after traumatic or adverse experiences, including earthquakes (21), cancer (22), suicide (23), and major infectious disease outbreaks (24). Research on positive psychological changes during and after the COVID-19 pandemic has been conducted in different populations, including the general public, youth, and people with COVID-19 (25, 26). Healthcare workers have received particular attention because of their direct involvement in pandemic response and their repeated exposure to infection risk, high workload, and emotional stress (11, 27–29). Studies in China have reported that 38.7 to 58.6% of healthcare workers experienced moderate-to-high levels of positive psychological changes during the pandemic (29–31). However, most existing studies were conducted during the early stage of the pandemic and mainly focused on hospital-based healthcare workers (32, 33). Less is known about positive psychological changes among CHWs in the post-pandemic period, despite their long-term participation in community-based prevention and control and their continued responsibility for primary health services. This gap is not only a difference in study population. CHWs had a different work setting, service relationship, and public health role from hospital-based healthcare workers, which may affect how pandemic-related stress was experienced, interpreted, and transformed into perceived psychological change (8, 10).

Factors associated with positive psychological changes among CHWs may include demographic characteristics (e.g., female gender, older age) (31, 34), psychosocial factors (e.g., self-efficacy, social support) (11, 29, 31), and COVID-19-specific factors (e.g., prior experience, working hours) (11, 29). Although rumination, coping strategies, and perceived social support have been widely examined in prior research on positive psychological changes, their roles may differ across populations and crisis contexts (34). For CHWs, these factors may be closely related to their community-based responsibilities, repeated contact with residents, changing work tasks, and support from families, colleagues, and organizations during and after the pandemic response. Examining these well-established predictors in this specific context can help assess whether existing evidence applies to primary care workers and can clarify how these factors are reflected in CHWs’ post-pandemic work experiences (35). Understanding these factors is important for designing public health and workplace support strategies that can protect the mental health of CHWs and strengthen the preparedness of primary care systems for future health emergencies (36). Based on previous theoretical and empirical evidence, we hypothesized that higher levels of positive coping, deliberate rumination, and perceived social support would be associated with greater positive psychological changes among CHWs after the COVID-19 pandemic. Therefore, this mixed-methods study aimed to assess the level of positive psychological changes and identify factors associated with positive psychological changes among CHWs in the post-pandemic period in China, and to further examine whether and how the quantitative findings related to rumination, coping strategies, and perceived social support were reflected in participants’ narratives through the qualitative phase (37–39).

## 2 Methods

### 2.1 Study design

This study used a mixed-methods design among community health workers (CHWs). In the first phase, a quantitative survey was conducted to assess the level of positive psychological changes and identify associated factors. In the second phase, semi-structured interviews were conducted to further understand CHWs’ experiences of these factors and to help explain the quantitative findings. Preliminary quantitative findings were used to inform the development of the qualitative interview guide, particularly findings related to positive coping, deliberate rumination, and family support. The qualitative findings were then compared with the quantitative results to explain how these factors were experienced by CHWs and how they may have contributed to positive psychological changes in the post-pandemic work context.

The study was conducted from April 29 to June 10, 2023, in Shenzhen, Guangdong Province, China, approximately three months after China announced the end of the latest wave of the COVID-19 pandemic. Shenzhen is a large city in southern China near Hong Kong SAR, with a population of more than 17 million. It has 783 community health centers (CHCs) and more than 12,000 CHWs. During the COVID-19 pandemic, Shenzhen experienced several waves of outbreaks and implemented national prevention and control policies. As in other regions of China, CHWs in Shenzhen were responsible for a wide range of COVID-19-related public health tasks, including nucleic acid testing, vaccination, health education, and community follow-up. At the same time, they continued to provide routine primary care services, such as managing residents’ health records, delivering basic medical services, and supporting chronic disease management. This setting provided an important opportunity to examine positive psychological changes among CHWs after a prolonged period of high work demand and direct involvement in community-based pandemic response.

### 2.2 Quantitative phase

#### 2.2.1 Participant recruitment and sample size

The quantitative phase was conducted from April 29 to June 10, 2023. CHCs were selected using district-stratified cluster sampling. Shenzhen has 10 administrative districts, and CHCs were selected from all 10 districts. Within each district, CHCs were selected according to the number of CHCs and CHWs in that district. After sample size calculation, the planned recruitment number was allocated proportionally according to the number of eligible CHWs in each district, and all eligible CHWs in selected CHCs were invited to participate.

A total of 51 CHCs were selected across all administrative districts of Shenzhen. The number of selected CHCs varied across districts, ranging from 1 to 12 centers per district. The number of eligible CHWs invited from each district ranged from 20 to 287. Across the 51 selected CHCs, the number of eligible CHWs per center ranged from 9 to 69, with a median of 22.0 (IQR, 18.0–31.5). These CHCs provided routine primary health care and public health services to local residents, including general outpatient care, chronic disease management, vaccination, health education, infectious disease prevention and control, and community follow-up (Supplementary Table S1).

Eligible participants included licensed physicians, assistant physicians, registered nurses, pharmacists, medical technicians, and other healthcare technical staff working in CHCs. CHWs who were mainly engaged in administrative or managerial work, as well as retired professionals, were excluded because they were not within the target population of active healthcare technical staff working in CHCs during the study period. The sample size for this quantitative study was determined using a cross-sectional study formula. Assuming a *Z-value* of 1.96 at a 95% confidence level, an allowable error (*d*) of 0.05, and a proportion (*p*) of 0.39 based on a previous study (27), the initial sample size was calculated as 366 participants. Additionally, considering the cluster sampling design, the sample size was increased by 50% (design effect = 1.5), resulting in an adjusted sample size of 549 participants. To ensure sufficient recruitment across multiple community health centers, the target sample size was rounded up to approximately 600 CHWs.

#### 2.2.2 Data collection

**Figure 1:**
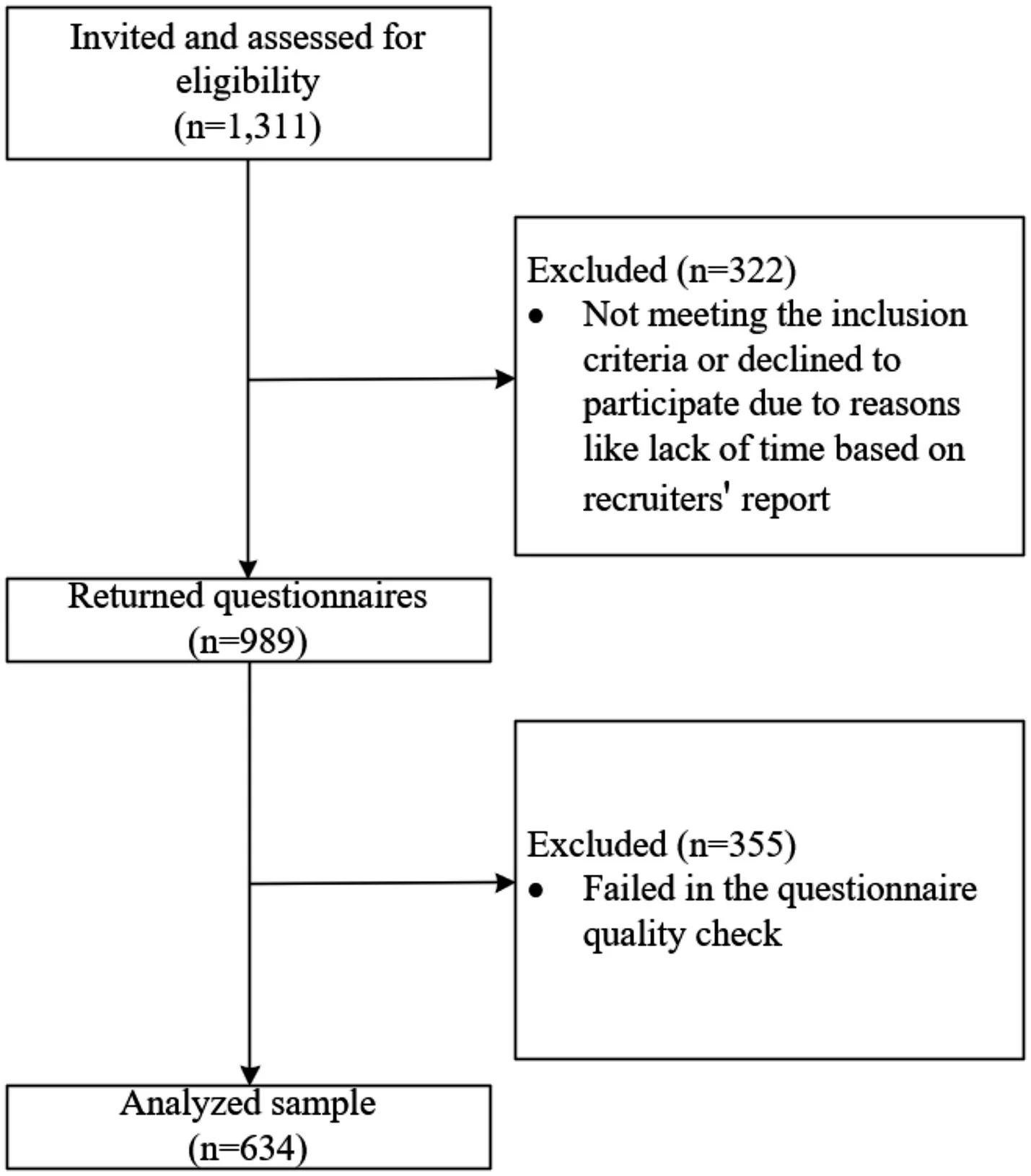
Flow chart of the quantitative study.

In the quantitative phase, an online self-administered survey was conducted using the Wenjuanxing platform. The questionnaire was distributed to eligible CHWs in the selected 51 CHCs through a QR code link. Before completing the questionnaire, participants were informed about the study purpose, voluntary participation, confidentiality, and their right to withdraw, and provided electronic informed consent. A total of 1,311 eligible CHWs were invited to participate, and 989 submitted questionnaires. After excluding 355 questionnaires due to incomplete responses, failed attention-check items, inconsistent responses, or implausibly short completion times, 634 valid questionnaires were included in the final analysis, exceeding the minimum required sample size of 600 (Figure 1). A comparison of baseline characteristics between included and excluded respondents showed no statistically significant differences (Supplementary Table S2).

#### 2.2.3 Measures

##### 2.2.3.1 Positive psychological change

Positive psychological change was measured using the 20-item Posttraumatic Growth Inventory Chinese version (C-PTGI). The original PTGI was a widely used 21-item questionnaire designed to assess perceived positive psychological changes after traumatic or highly stressful experiences across five domains: relating to others, new possibilities, personal strength, spiritual change, and appreciation of life. Consistent with previous studies in China, the C-PTGI used in the present study was culturally adapted to a 20-item version by removing the item “I have a stronger religious faith” (29, 32, 40, 41). Each item is rated on a 6-point Likert scale (0 = no change to 5 = complete change), with total scores ranging from 0 to 100. A summative score was calculated, with higher scores indicating greater levels of positive psychological change. In this study, the C-PTGI showed high internal consistency, with Cronbach’s *α* of 0.96 for the overall scale and *α* values for subdomains ranging from 0.82 to 0.90.

##### 2.2.3.2 Rumination

Participants’ rumination was assessed using the Event-Related Rumination Inventory (ERRI) (42), and it was validated in Chinese healthcare workers (29, 43). The 20-item ERRI includes two subscales: intrusive rumination and deliberate rumination. Items are scored on a 4-point Likert scale from 0 (“I never think of this after the experience”) to 3 (“I often think of this after the experience”). Total scores range from 0 to 60, with subscale scores ranging from 0 to 30. Higher scores indicate greater levels of rumination. The Cronbach’s *α* for intrusive rumination and deliberate rumination was 0.95 and 0.94 in this study, respectively.

##### 2.2.3.3 Coping strategy

The Trait Coping Style Questionnaire (TCSQ) was used to assess coping strategies, specifically evaluating positive and negative coping styles individuals may adopt when facing life-threatening experiences (44). Positive coping refers to active and constructive responses to stressful events, such as problem-solving, seeking support, reframing difficulties, and maintaining confidence in managing challenges. Negative coping refers to passive or avoidant responses, such as withdrawal, denial, or emotional venting without problem-solving. The scale consists of 20 items, each rated on a 5-point Likert scale ranging from 1 (strongly disagree) to 5 (strongly agree), with higher scores in each dimension indicating a greater tendency to use that coping style. The Chinese version of the TCSQ has been validated among Chinese healthcare workers (45, 46), with Cronbach’s *α* of 0.83 for negative coping and 0.96 for positive coping in this study.

##### 2.2.3.4 Social support

Social support was measured by the Multidimensional Scale of Perceived Social Support (PSSS) (47). The Chinese version of the PSSS has been validated among healthcare workers in China (48). The scale consists of 12 items rated on a 7-point Likert scale ranging from 1 (strongly disagree) to 7 (strongly agree) and is divided into three domains: family support, friend support, and significant other support, with higher scores indicating greater social support. In this study, the overall Cronbach’s *α* of the PSSS was 0.95, with subscales ranging from 0.90 to 0.94 in this study.

##### 2.2.3.5 Variables related to COVID-19

Participants were asked about their experiences related to COVID-19, including whether they had any acquaintances or family members who passed away due to COVID-19 (Yes/No), whether they had experienced work-related quarantine (Yes/No), and whether they had ever been infected with COVID-19 (Yes/No).

##### 2.2.3.6 Background variables

Background information included age (in years), gender (Male/Female), marital status (Married/Living with a partner/Unmarried/Divorced/Separated/Widowed), education level (College and below/Master and above), monthly income (≤10,000 CNY/>10,000 CNY), occupation (Doctor/Nurse/Other CHW), professional title (Primary title/Junior title/Senior title).

#### 2.2.4 Data analysis

Descriptive analyses were first conducted for all study variables. Continuous variables were summarized using means and standard deviations or medians and interquartile ranges, as appropriate, and categorical variables were summarized using frequencies and percentages. Because the main outcome was defined as the presence of moderate-to-high positive psychological change, logistic regression was used for the association analyses. Following previous studies, participants with a mean item score of ≥3 in at least one PTGI domain were classified as having moderate-to-high positive psychological change (11, 49), indicating that they reported at least a moderate degree of perceived positive change in one or more PTGI domains. Participants who did not meet this criterion were classified as having lower positive psychological change. This operational definition was used to describe participants reporting at least moderate perceived positive change rather than as a validated clinical cutoff.

Univariate logistic regression analyses were first performed to examine the crude associations between each independent variable and moderate-to-high positive psychological change. Multivariable logistic regression was then used to identify factors associated with moderate-to-high positive psychological change after adjustment for other variables in the model. Variables entered into the multivariable model were selected based on the revised PTG model, previous literature, and the study objectives, including demographic characteristics, COVID-19-related exposure variables, rumination, coping strategies, and perceived social support. Because this study aimed to identify the main associated factors rather than test effect modification, interaction terms were not included in the final model. The crude odds ratio (OR), adjusted odds ratio (AOR), and 95% confidence interval (CI) were reported. Given the cross-sectional design, regression results were interpreted as associations rather than causal effects. Analyses were conducted using available data without imputation. A two-sided *p-value* <0.05 was considered statistically significant. All analyses were performed using R, version 4.3.2.

To assess whether the findings were sensitive to dichotomizing positive psychological change, we conducted a sensitivity analysis using the continuous total score as the outcome. Univariate and multivariable linear regression models were fitted using the same set of independent variables included in the logistic regression models. The sensitivity analysis was used to examine whether the overall pattern of associated factors was consistent with the primary analysis.

### 2.3 Qualitative phase

#### 2.3.1 Participant recruitment and sample size

Eligible participants for the qualitative phase included CHWs working in Shenzhen CHCs, including licensed physicians, assistant physicians, registered nurses, pharmacists, medical technicians, and other healthcare technical staff. CHWs who were mainly engaged in administrative or managerial work, as well as retired professionals, were excluded. Purposive sampling was used to recruit participants who could provide rich information about their work experiences during the COVID-19 pandemic and their perceived psychological changes after the pandemic. To increase variation in participants’ experiences, the sampling process considered occupation, gender, age, professional title, and positive psychological change level based on the preliminary quantitative results. In total, 12 CHWs were interviewed. This sample size was considered appropriate for interpretative phenomenological analysis (IPA), which focuses on the detailed examination of individual lived experiences and comparison across cases rather than statistical representativeness (50). Recruitment and preliminary analysis were conducted iteratively. Interviews continued until no new major themes or meaning units emerged from the data, suggesting that data saturation had been reached.

#### 2.3.2 Data collection

A semi-structured interview guide with open-ended questions was developed before data collection and refined based on the preliminary quantitative findings (Supplementary Table S3). These questions explored how CHWs used positive coping strategies in response to pandemic-related stress, how they reflected on and made sense of their experiences, and how family support and other forms of social support influenced their psychological adjustment. The interviews also asked participants to describe concrete examples of growth, including changes in personal strength, relationships with others, appreciation of life, new possibilities, and reflections on life. Two pilot interviews were conducted before formal data collection to assess the clarity, relevance, and openness of the interview questions. The interview guide was revised based on feedback from the pilot interviews. Formal interviews were conducted by two researchers from the study team (one male and one female) who had previous experience in qualitative research. Before data collection, both interviewers received training on semi-structured interviewing techniques, probing strategies, and the principles of IPA. The interviewers had no prior relationship with the participants and were not involved in their workplace management or evaluation.

During the interviews, interviewers used follow-up questions when needed to clarify participants’ responses and obtain more detailed information. Each interview lasted approximately 90 to 120 min and was audio-recorded with participants’ permission. Written informed consent was obtained from each participant before the interview. Before data collection, the research team recognized that positive psychological change represented one possible interpretation of pandemic-related experiences rather than an inevitable outcome of adversity. Researchers remained open to diverse experiences and interpretations, including both positive and negative psychological responses, throughout data collection and analysis. Reflexive discussions were conducted throughout the analytic process to consider how researchers’ backgrounds and perspectives might influence the interpretation of participants’ narratives.

#### 2.3.3 Data analysis

Interview data were analyzed using IPA to explore how CHWs interpreted their pandemic-related experiences and perceived psychological changes. Audio recordings were transcribed verbatim, and transcripts were reviewed repeatedly to achieve familiarity with each participant’s narrative. Following the idiographic principles of IPA, each transcript was first analyzed individually as a separate case. Researchers conducted detailed coding of meaningful statements, focusing on participants’ experiences of pandemic-related work, perceived psychological changes, coping processes, cognitive reflection, and social support. Individual case summaries were developed to capture each participant’s unique experiences and interpretations before moving to cross-case analysis.

After completing the individual case analyses, researchers compared patterns across cases to identify shared experiential themes and variations between participants. Two researchers independently coded the transcripts and discussed their interpretations throughout the analytic process. Disagreements were resolved through discussion, and a third researcher was consulted when necessary. Related themes were then clustered to develop an interpretation of CHWs’ positive psychological change experiences and the factors that shaped these experiences, while maintaining attention to individual meanings. Representative quotations were selected to illustrate each theme. After the preliminary themes and interpretations were developed, the findings were returned to participants for verification. Participants were invited to provide feedback on whether the interpretations accurately reflected their experiences, and their feedback was considered during the finalization of the qualitative findings. NVivo version 12 was used to support data management and coding.

### 2.4 Integration of quantitative and qualitative findings

The integration of quantitative and qualitative findings occurred at three stages. First, preliminary quantitative results were used to refine the qualitative interview guide, with particular attention to factors associated with positive psychological change, including positive coping, deliberate rumination, and family support. Second, during qualitative analysis, themes were compared with the quantitative findings to examine how participants’ narratives supported, explained, or differed from the statistical associations. Third, the integrated findings were interpreted together in the Discussion to provide a more complete understanding of positive psychological change among CHWs in the post-pandemic context.

## 3 Results

### 3.1 Quantitative results

#### 3.1.1 Participant characteristics and level of positive psychological change

A total of 634 CHWs were included in the quantitative analysis. The mean age of the participants was 35.7 years (SD = 8.2). Most participants were female (*n* = 482, 76.0%), married or living with a partner (*n* = 444, 70.0%), and had an undergraduate degree or above (*n* = 570, 89.9%). Detailed participant characteristics are shown in Table 1.

**Table 1:** Distributions of background variables and positive psychological change of this study.

Variables	Overall (*N* = 634)	Lower positive psychological change (*n* = 261)	Moderate to high positive psychological change (*n* = 373)
	*N* (%)/Mean±SD		
Social demographic variables			
Age	35.7 ± 8.2	35.6 ± 8.0	35.8 ± 8.3
18–25	47 (7.4)	20 (3.2)	27 (4.2)
26–35	305 (48.1)	124 (19.6)	181 (28.5)
36–45	190 (30.0)	78 (12.3)	112 (17.7)
46–55	83 (13.1)	37 (5.8)	46 (7.3)
>55	9 (1.4)	2 (0.3)	7 (1.1)
Gender			
Male	152(24.0)	61 (9.6)	91 (14.4)
Female	482(76.0)	200 (31.5)	282 (44.5)
Marital status			
Married/living with partner	444 (70.0)	182 (28.7)	262 (41.3)
Unmarried/divorced/separated/widowed	190 (30.0)	79 (12.5)	111 (17.5)
Monthly income (CNY)^a^			
<5,000	29 (4.6)	11 (1.7)	18 (2.8)
5,001-8,000	109 (17.2)	39 (6.2)	70 (11.0)
8,001–10,000	159 (25.2)	76 (12.0)	83 (13.1)
10,001-15,000	208 (32.8)	81 (12.8)	127 (20.0)
15,001-20,000	80 (12.6)	36 (5.7)	44 (6.9)
>20,000	49 (7.7)	18 (2.8)	31 (4.9)
Education level			
Technical secondary school or junior college	64 (10.1)	21 (3.3)	43 (6.8)
Undergraduate	508 (80.1)	212 (33.4)	296 (46.7)
Master degree and above	62 (9.8)	28 (4.4)	34 (5.4)
Occupation			
Doctor	308 (48.6)	128 (20.2)	180 (28.4)
Nurse	228 (35.9)	100 (15.8)	128 (20.2)
Others^b^	98 (15.5)	33 (5.2)	65 (10.3)
Professional title^c^			
Primary title	218 (34.4)	88 (13.9)	130 (20.5)
Junior title	358 (56.5)	150 (23.7)	208 (32.8)
Senior title	58 (9.1)	23 (3.6)	35 (5.5)
Variables related to COVID-19			
Have any acquaintances or family members passed away due to COVID-19?			
Yes	146 (23.0)	58 (9.1)	88 (12.9)
No	488 (77.0)	203 (32.0)	285 (45.0)
Have you ever experienced quarantine due to work?			
Yes	194 (30.6)	62 (9.8)	132 (20.8)
No	440 (69.4)	199 (31.4)	241 (38.0)
Have you ever been infected with COVID-19?			
Yes	512 (80.8)	204 (32.2)	308 (48.6)
No	122 (19.2)	57 (9.0)	65 (10.2)
Psycho-social variables			
Coping			
Negative coping	27.3 ± 6.9	28.0 ± 6.4	26.8 ± 7.2
Positive coping	33.0 ± 5.7	30.7 ± 5.6	34.7 ± 5.3
Rumination			
Intrusive rumination	5.5 ± 5.3	5.3 ± 5.3	5.5 ± 5.4
Deliberate rumination	8.0 ± 5.5	6.9 ± 5.4	8.7 ± 5.4
Social support			
Family support	21.5 ± 4.9	19.8 ± 4.8	22.7 ± 4.5
Friend support	21.0 ± 4.3	19.4 ± 4.3	22.1 ± 4.0
Significant other support	20.1 ± 4.5	18.6 ± 4.2	21.1 ± 4.4

^a^ CNY, Chinese yuan, 1USD ≈ 7CNY.

^b^ Others include pharmacists, imaging technicians, and other healthcare technical personnel.

^c^ Primary titles include community health workers at both the primary titles and those without specific titles. Senior titles encompass community health workers holding associate senior titles as well as those with senior titles.

**Figure 2:**
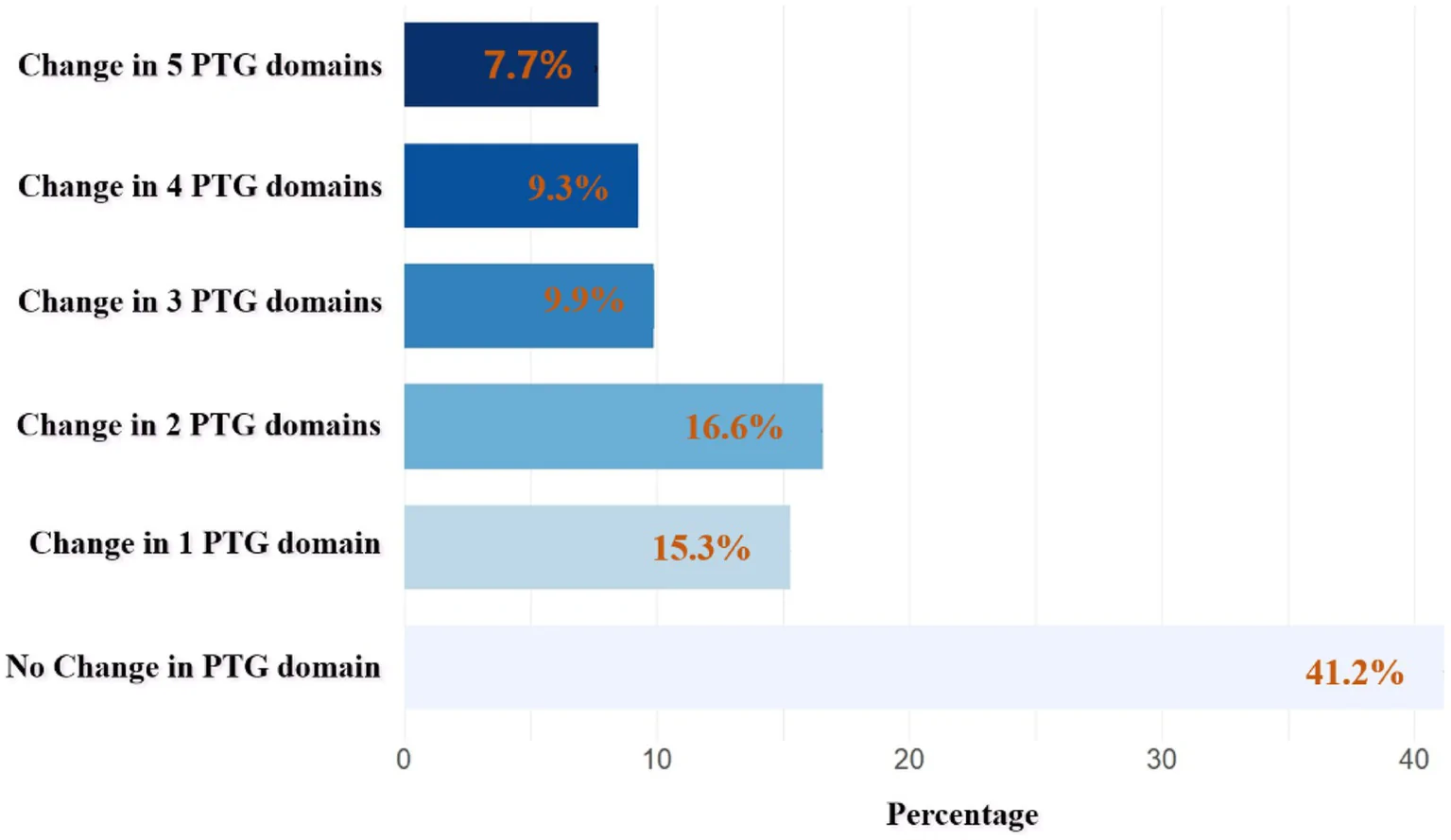
Percentage of positive psychological change in at least one dimension among CHWs in China.

As shown in Figure 2, 58.8% of participants (*n* = 373) reported moderate-to-high positive psychological change in at least one PTGI domain. Specifically, 15.3, 16.6, 9.9, 9.3, and 7.7% of participants reported moderate-to-high positive psychological change in 1, 2, 3, 4, and 5 domains, respectively. Thus, 43.5% of participants reported moderate-to-high positive psychological change in two or more domains, 26.9% in three or more domains, and 17.0% in four or more domains.

#### 3.1.2 Factors associated with moderate-to-high positive psychological change

The results of the univariate and multivariable logistic regression analyses are shown in Table 2. In the univariate analyses, higher positive coping, higher deliberate rumination, higher family support, higher friend support, higher significant other support, and work-related quarantine experience due to COVID-19 were associated with higher odds of moderate-to-high positive psychological change. In contrast, higher negative coping was associated with lower odds of moderate-to-high positive psychological change.

**Table 2:** Univariate and multivariable logistic regression analyses of positive psychological change and selected variables (*N* = 634).

Independent variables	Moderate to high positive psychological change			
	Crude OR	95%CI	Adjusted OR	95%CI
Social demographic variables				
Age (years)	1.00	(0.98, 1.02)	0.99	(0.97, 1.02)
Gender (female versus male)	0.95	(0.65, 1.37)	0.81	(0.49, 1.33)
Marital status (married versus unmarried)	1.02	(0.73, 1.45)	0.78	(0.49, 1.25)
Education level (master or above versus college or below)	0.83	(0.49, 1.41)	0.71	(0.36, 1.40)
Monthly income (>10,000 versus ≤10,000 CNY)	1.10	(0.80, 1.51)	1.05	(0.71, 1.55)
Occupational type (reference: others)				
Nurse	0.65	(0.40, 1.06)	0.78	(0.44, 1.41)
Doctor	0.71	(0.44, 1.15)	0.74	(0.41, 1.34)
Technical qualification (junior title and above versus primary title)	0.95	(0.68, 1.33)	0.98	(0.60, 1.59)
Variables related to COVID-19				
Families died due to COVID-19 (Yes versus No)	1.08	(0.74, 1.58)	0.83	(0.54, 1.29)
Quarantine experience due to COVID-19 (Yes versus No)	1.76**	**(1.23, 2.51)**	1.43	(0.96, 2.13)
COVID-19 infection	1.32	(0.89, 1.97)	1.24	(0.78, 1.95)
Psycho-social variables				
Negative coping (score)	0.97*	**(0.95, 0.99)**	1.00	(0.97, 1.04)
Positive coping(score)	1.16***	**(1.12, 1.20)**	1.13***	**(1.08, 1.18)**
Intrusive rumination (score)	1.01	(0.98, 1.04)	0.95	(0.90, 1.01)
Deliberate rumination (score)	1.07***	**(1.04, 1.10)**	1.12***	**(1.06, 1.18)**
Family support (score)	1.14***	**(1.10, 1.18)**	1.08**	**(1.02, 1.14)**
Friend support (score)	1.17***	**(1.13, 1.22)**	1.07	(0.99, 1.14)
Significant other support (score)	1.14***	**(1.10, 1.19)**	0.99	(0.93, 1.06)

* *p* < 0.05; **p < 0.01;***p < 0.001. Bold values indicate statistically significant associations (*p* < 0.05).

After adjustment for demographic characteristics, COVID-19-related exposure variables, rumination, coping strategies, and perceived social support, moderate-to-high positive psychological change remained significantly associated with higher positive coping (AOR = 1.13, 95% CI: 1.08–1.18), higher deliberate rumination (AOR = 1.12, 95% CI: 1.06–1.18), and higher family support (AOR = 1.08, 95% CI: 1.02–1.14). Negative coping, work-related quarantine experience, friend support, and significant other support were significant in the univariate analyses but were no longer statistically significant after adjustment. Other demographic and COVID-19-related variables were not significantly associated with moderate-to-high positive psychological change in the multivariable model. The multivariable logistic regression model showed adequate goodness of fit (Hosmer–Lemeshow test: *p* = 0.387), with a Nagelkerke R^2^ of 0.271. All VIF values were below 2.5.

In the sensitivity analysis using the continuous total score as the outcome, positive coping, deliberate rumination, and family support remained positively associated with positive psychological change in the multivariable linear regression model. Work-related quarantine and friend support were also positively associated with the continuous positive psychological change (Supplementary Table S4).

### 3.2 Qualitative results

#### 3.2.1 Participant characteristics

The qualitative phase included interviews with 12 CHWs from 6 CHCs. To protect participants’ privacy, participants were identified using codes A–L. Among them, 8 were female (66.7%), 6 were doctors (50.0%), and the mean age was 35.3 years (Supplementary Table S5).

#### 3.2.2 Qualitative findings of positive psychological change

Following the idiographic principles of IPA, each interview transcript was first analyzed as an individual case to explore participants’ lived experiences and interpretations of pandemic-related events. Cross-case analysis was then conducted to identify shared experiential patterns across participants. Five themes were identified: enhanced personal strength, changes in interpersonal relationships, greater appreciation of life, new possibilities in life, and reflections on life and values. These themes are summarized in Table 3.

**Table 3:** Themes, subthemes, and representative quotations of positive psychological change in the qualitative phase.

Theme	Subtheme	Sentence
Enhanced personal strength	Professional knowledge	*“Before, when seeing patients with fever or cough, you might think of some upper respiratory infections or some basic diseases. Now, when you see these patients, you will be very cautious, wondering if there are some other diseases you have not discovered. Definitely, there will be some improvements in this aspect (G, female, doctor).”*
	Comprehensive abilities	*“At the beginning, I was at a loss, but now, I am not so afraid and can handle it more calmly. This is because with more experience, you become more confident (E, male, doctor).”*
		*“I think if there are emergencies again, our whole team might be more experienced, or say, have a better mindset (H, female, nurse).”*
Changes in interpersonal relationships	Departmental cooperation	*“(Before the COVID-19 pandemic) We did not have such close contact with the workstations, and even less so with the police. Now, we need to be in contact with many departments (A, female, nurse).”*
		*“For some community residents with mobility issues, (community workers) have a clearer idea. They help us locate these people and then take us there. Also, in private matters, like if I get something lost, asking for their help (J, female, other CHW).”*
	Doctor-patient relationships.	*“I feel that patients will have higher trust and reliance on primary healthcare…. We support each other. Through the family doctor WeChat group, they ask doctors if they have any problems. So, in this aspect, I feel that the public’s trust in primary healthcare doctors will be higher (G, female, doctor).”*
Appreciations of life	Valuing health	*“Now I just feel that health is first, life is first, living is first. Being able to live healthily and happily, this is really not easy (H, female, nurse).”*
	Spending more time with family	*“I feel like I should spend more time with my family and children…. Now, looking back, if this pandemic had not happened, I would not have done this. I would have gone out to play…. I think I’m more family-oriented now (J, female, other CHW).”*
	Cherishing life	*“I cherish more those days when you can just go wherever you want. When I have a holiday, I might choose to travel or do something like that. Compared to the time during the pandemic, and even before the pandemic, I cherish it more now (L, female, nurse).”*
New possibilities in life	Healthy lifestyle	*“Now, after it’s over, I feel that I do not have to work overtime every night, and recently I have been playing badminton with my husband. I exercise more than before (A, female, nurse).”*
		*“Previously, I might have done less in this aspect. Now, I pay more attention, including dietary adjustments and exercise, and overall physical quality adjustment, yes, I value these things more (B, female, doctor).”*
	Leisure activities	*“During the pandemic, the pressure was quite high, and I often went to parks and played around. The pressure was too much. Now, when I need to relax, I go out to play, which has become a new interest (E, male, doctor).”*
Reflections on life		*“In the past, I might have felt a bit guilty because of other people’s misunderstandings, but not anymore. I will not feel guilty because I know I was trying to help them (I, male, other CHW).”*
		*“It’s not easy to live. We saw cases like Hong Kong people coming back to visit their families. After their father passed away, they still had to be quarantined, and they did not even get to see him for the last time. So, I value life more (B, female, doctor).”*
		*“Because I’ve seen a lot of reports or experienced it myself, I feel that life can be lost too easily…. Now, I think I cherish the present life more, and that perception and satisfaction of happiness, I find it in very ordinary things, which I did not before. I think this is the biggest change (K, male, doctor).”*

##### 3.2.2.1 Enhanced personal strength

Participants described enhanced personal strength after working through the COVID-19 pandemic. This theme referred mainly to increased professional confidence, improved ability to respond to public health emergencies, and a stronger sense of competence in managing complex work tasks. Some participants reported that they felt uncertain and anxious at the beginning of the pandemic, but became more confident as they gained experience in pandemic response.

"At the beginning, I was at a loss, but now, I am not so afraid and can handle it more calmly. This is because with more experience, you become more confident (E, male, doctor)."

##### 3.2.2.2 Changes in interpersonal relationships

Participants described positive changes in interpersonal relationships. These changes included closer collaboration among colleagues, stronger cross-departmental cooperation, and improved relationships between CHWs and residents. Some participants felt that the pandemic made residents more aware of the role of primary healthcare and increased their trust in CHWs.

"I feel that patients will have higher trust and reliance on primary healthcare… We support each other. Through the family doctor WeChat group, they ask doctors if they have any problems. So, in this aspect, I feel that the public's trust in primary healthcare doctors will be higher (G, female, doctor)."

##### 3.2.2.3 Greater appreciation of life

Participants commonly described a greater appreciation of life after the COVID-19 pandemic. This theme focused on valuing health, spending more time with family, and cherishing ordinary daily life. Participants often linked this change to their direct work experience during the pandemic and their increased awareness of uncertainty in life.

"I feel like I should spend more time with my family and children… Now, looking back, if this pandemic hadn't happened, I wouldn't have done this. I would have gone out to play… I think I’m more family-oriented now (J, female, other CHW)."

##### 3.2.2.4 New possibilities in life

Participants described how pandemic experiences led them to consider new possibilities in life. This theme included adopting healthier lifestyles, paying more attention to physical health, and making changes in leisure activities and daily routines. These changes were not limited to work but also reflected broader adjustments in how participants planned and organized their lives.

"Previously, I might have done less in this aspect. Now, I pay more attention, including dietary adjustments and exercise, and overall physical quality adjustment, yes, I value these things more (B, female, doctor)."

##### 3.2.2.5 Reflections on life and values

Participants described deeper reflections on life and values after the pandemic. Unlike the theme of greater appreciation of life, which focused mainly on cherishing health, family, and daily life, this theme reflected broader meaning-making and changes in how participants understood life, work, happiness, and personal priorities. Some participants reported that the pandemic made them re-evaluate what they considered important.

"Because I have seen many reports and experienced it myself, I feel that life can be lost too easily… Now, I cherish the present life more, and I find happiness in very ordinary things, which I did not before. I think this is the biggest change (K, male, doctor)."

#### 3.2.3 Qualitative findings of factors associated with positive psychological change

Through cross-case analysis of individual participant narratives, four experiential themes related to factors associated with positive psychological change were identified: personal resources, social support, coping strategies, and cognitive processing. These themes are summarized in Supplementary Table S6.

##### 3.2.3.1 Personal resources

Participants described personal resources as important for adjusting to pandemic-related work stress. These resources included professional responsibility, emotional control, work commitment, and confidence gained through repeated involvement in pandemic response tasks. Participants reported that these resources helped them maintain their work role and manage negative emotions during periods of high pressure.

"We try our best to leave negative emotions aside at work and stay as energetic as possible. This was almost always the case, even during the hardest times (H, female, nurse)."

##### 3.2.3.2 Social support

Social support was repeatedly described as an important source of psychological adjustment. Participants mentioned support from family members, colleagues, residents, and institutions. Family support was especially important because it reduced family–work conflict and allowed CHWs to focus on demanding pandemic-related tasks.

"My family is quite supportive because they understand the nature of my work and the unique demands of this era. Fortunately, as I only have one child, both my parents and my in-laws help out. They take care of my child, so I don't have to worry about home issues, which really alleviates a lot of my concerns (C, female, doctor)."

##### 3.2.3.3 Coping strategies

Participants described different coping strategies during the pandemic. Positive coping was reflected in actively facing work demands, regulating emotions, seeking practical ways to reduce stress, and continuing to perform professional responsibilities despite uncertainty. Some participants also described passive acceptance and helplessness, suggesting that coping responses were not always positive.

"No matter how difficult things are, being afraid is useless. When it comes, you have to face it… If the pressure is too much, just go out for a meal and do some shopping. For us, shopping can also alleviate some stress (A, female, nurse)."

Some participants also described a more passive response to pandemic-related work pressure:

"You can only do this, you have no choice but to do it (L, female, nurse)."

##### 3.2.3.4 Cognitive processing

Cognitive processing reflected how participants repeatedly thought about pandemic-related experiences and tried to understand their meaning. Participants described recalling memorable events, comparing the most difficult periods with the present, and rethinking their work, relationships, and life priorities. These reflections were often linked to perceived changes in personal strength, appreciation of life, and professional identity.

"From the beginning to the end, we were fully involved. Of course, we went through many things, so some particularly unforgettable incidents sometimes come to mind, making me feel that it was a very special experience…. Sometimes, when I think about a certain event, I realize how we were back then and how much better the situation is now (C, female, doctor)."

### 3.3 Integration of quantitative and qualitative findings

The qualitative findings helped explain and extend the quantitative results. In the quantitative phase, 58.8% of CHWs reported moderate-to-high positive psychological change. Consistent with this finding, the qualitative interviews showed that participants described positive psychological changes across five areas: enhanced personal strength, changes in interpersonal relationships, greater appreciation of life, new possibilities in life, and reflections on life and values. These qualitative themes provided concrete examples of how positive psychological change was experienced in the post-pandemic work context of CHWs. Importantly, these qualitative themes were not separate from participants’ COVID-19-related work experiences. Participants linked perceived positive changes to specific features of the pandemic response, including prolonged work pressure, uncertainty during community-based prevention and control, frequent contact with residents, and the need to maintain routine primary care while undertaking additional public health tasks. These accounts suggested that positive psychological change among CHWs was shaped by the specific occupational context of the COVID-19 pandemic, rather than by exposure to a single acute traumatic event alone.

The integration of findings also clarified the factors associated with positive psychological change. Quantitatively, higher positive coping, deliberate rumination, and family support were significantly associated with moderate-to-high positive psychological change after adjustment. Qualitatively, participants described positive coping as actively facing work demands, regulating emotions, and seeking practical ways to manage stress. Their narratives also showed that cognitive processing involved repeated reflection on pandemic-related experiences and reinterpretation of these experiences over time, which helped explain the quantitative association between deliberate rumination and positive psychological change. In addition, family support was described as reducing family–work conflict and helping CHWs maintain their work responsibilities during the pandemic, which supported the quantitative finding that family support was associated with moderate-to-high positive psychological change.

Some findings were complementary rather than fully consistent. Friend support, significant other support, work-related quarantine experience, and negative coping were associated with positive psychological change only in the univariate analyses, but not after adjustment. The qualitative interviews suggested that support from colleagues, residents, and institutions still played a role in CHWs’ psychological adjustment, although family support appeared more directly connected to their ability to manage work and family demands. Similarly, participants described both positive coping and passive acceptance, suggesting that different coping responses may coexist during prolonged public health emergencies. Taken together, the mixed-methods findings suggest that positive psychological change among CHWs after the COVID-19 pandemic was related not only to individual coping and cognitive processing, but also to the family and work context in which these psychological changes occurred.

## 4 Discussion

Positive psychological change signifies positive psychological transformations in individuals following traumatic experiences. This mixed-methods study adds evidence from a primary care workforce that has received less attention than hospital-based healthcare workers by focusing on CHWs, who maintained routine primary care while undertaking community-based COVID-19 response tasks. Furthermore, it establishes a solid foundation for enhancing CHWs’ mental health in response to future abrupt public health incidents.

The revised model of PTG was used as a guiding framework in this study, but the COVID-19 pandemic had several contextual features that needed to be considered when applying this model. Unlike a single acute traumatic event, the pandemic was prolonged, collective, and partly uncontrollable. For CHWs, it was closely tied to infection risk, changing public health tasks, resident contact, family-work pressure, and the continued need to provide routine primary care. These experiences may have challenged assumptions about safety, control, professional role, health, family life, and the value of primary care work. Within this context, coping strategies, deliberate rumination, and social support may have shaped how CHWs managed stress, reflected on their experiences, and reconstructed meaning after the most intense phase of the pandemic response.

The finding that more than half of CHWs reported moderate-to-high positive psychological change indicates that positive psychological changes were common in this workforce group after a prolonged period of pandemic-related work pressure. Compared with studies among resident doctors in China, nurses in Hong Kong and Taiwan, psychiatric physicians in the United Kingdom, and healthcare workers across mainland China, the proportion of participants with moderate-to-high positive psychological change in this study appeared relatively high (27, 31, 51, 52). One possible explanation is the timing of data collection. This study was conducted approximately three months after China announced the end of the latest wave of the COVID-19 pandemic, which may have allowed CHWs more time to reflect on their pandemic-related experiences. According to the revised model of PTG, positive psychological change may develop through emotional struggle, cognitive processing, and meaning reconstruction over time (34). However, these comparisons should be interpreted with caution because studies differed in population, measurement, timing, local pandemic conditions, and work roles.

Among the PTG dimensions, appreciation of life was the most commonly reported domain, which is consistent with previous research among healthcare workers (31). The qualitative findings helped explain this result. Participants described paying more attention to health, spending more time with family, and finding value in ordinary daily life after the pandemic. These findings suggest that close contact with illness, uncertainty, and intense community-based work may have changed how CHWs understood health, family, and daily life. In contrast, the spiritual change domain was less prominent. This may partly reflect cultural differences in religious belief and spiritual expression in China. In addition, because the culturally adapted PTGI used in this study removed the religious faith item, findings related to this domain should be interpreted with caution. The qualitative theme of reflections on life and values partly captured meaning-making beyond religious belief, suggesting that CHWs may express existential reflection in ways that are not fully measured by the original spiritual change items.

Regarding factors associated with positive psychological change, the quantitative and qualitative findings can be interpreted in relation to the revised model of PTG, while recognizing the cross-sectional nature of the data. In this model, coping strategies, deliberate rumination, and social support are closely related to how individuals manage stressful experiences, reflect on their meaning, and draw on interpersonal resources during psychological adjustment. However, because all variables were measured at a single time point, these associations should not be interpreted as evidence that coping strategies, deliberate rumination, or social support caused positive psychological change or preceded its development. Reverse or bidirectional relationships are also possible. For example, CHWs who reported higher positive psychological change may have been more likely to recall, interpret, or report their coping and reflective processing positively.

Positive coping was significantly associated with moderate-to-high positive psychological change in the quantitative analysis, and this association was supported by the qualitative findings, aligning with previous research (11, 53, 54). Participants described positive coping as actively facing work demands, regulating negative emotions, using practical stress relief strategies, and maintaining professional responsibility during difficult periods. These narratives suggest that positive coping was closely related to CHWs’ descriptions of active adjustment rather than passive endurance when facing prolonged public health stress. This interpretation is consistent with PTG theory, which links coping responses with cognitive processing and meaning reconstruction after stressful experiences (53). However, this finding should be interpreted in relation to the specific context of the COVID-19 pandemic. The pandemic was a prolonged and partly uncontrollable public health crisis, and the role of coping strategies may have depended on work demands, perceived control, organizational support, and available resources. Therefore, although the term positive coping was retained from the original TCSQ subscale, it should not be interpreted as meaning that active coping strategies are always beneficial across all contexts. The interviews also showed that positive and negative coping could coexist. Some participants described passive acceptance or helplessness, such as feeling that they “had no choice” but to continue their work. In the quantitative analysis, negative coping was associated with lower odds of moderate-to-high positive psychological change only in the univariate analysis and was not significant after adjustment. This pattern suggests that the role of negative coping may be partly linked to other psychosocial factors, such as social support or rumination. It also indicates that coping strategies labeled as negative in the TCSQ may not have a simple relationship with positive psychological change in a prolonged public health crisis. Future studies should further examine how the timing, duration, and perceived controllability of stressful experiences shape the relationship between coping strategies and positive psychological change among CHWs.

Family support was also significantly associated with moderate-to-high positive psychological change after adjustment. This finding was strongly supported by the qualitative interviews (55, 56). Participants described family support as helping them manage childcare, household responsibilities, and emotional pressure during periods of intense work. For CHWs, who often had to balance routine primary care with COVID-19-related community tasks, family support may have reduced family–work conflict and allowed them to continue their professional duties with less worry. Although friend support and significant other support were significant only in the univariate analyses, the qualitative findings showed that broader support from colleagues, residents, and institutions still mattered for psychological adjustment. This difference suggests that different sources of support may have different roles. Family support may be more directly linked to daily role balance, while colleague and institutional support may shape the broader work environment (57).

Deliberate rumination was another factor significantly associated with moderate-to-high positive psychological change, aligning with findings from previous studies (11, 29, 58). The qualitative findings showed that CHWs often recalled specific pandemic-related events, compared the most difficult periods with the present, and reflected on what these experiences meant for their work and life. This kind of cognitive processing was not simply repetitive distress. Rather, participants described a more purposeful process of reviewing past experiences, recognizing changes, and rethinking life priorities. This finding is consistent with the revised model of PTG, which links deliberate rumination with meaning reconstruction after major stressors (59). It also helps explain why the qualitative themes included both appreciation of life and reflections on life and values. Appreciation of life reflected more direct changes in valuing health, family, and daily living, whereas reflections on life and values reflected broader meaning-making about work, happiness, and personal priorities.

### 4.1 Implications for interventions promoting positive psychological change among CHWs

This study has practical implications for supporting CHWs after public health emergencies. First, workplace mental health programs for CHWs could include coping support, stress management, emotional regulation, and structured opportunities for CHWs to discuss difficult work experiences (60). These components are consistent with previous evidence that coping strategies, resilience, and social support are related to psychological outcomes among healthcare workers during the COVID-19 pandemic (61). Second, family-sensitive support is important. Flexible scheduling, childcare support, family communication resources, and attention to family–work conflict may help reduce the pressure faced by CHWs during emergency response (62, 63). Third, organizations should create safe and supportive spaces for CHWs to reflect on pandemic-related work experiences, share work stories, and receive support from supervisors and colleagues. Such measures may help CHWs process stressful experiences and make sense of their work role after emergency response (64, 65).

### 4.2 Limitations

This study has several limitations. First, the cross-sectional design limits causal inference. Although the study was guided by the revised model of PTG and identified several factors associated with moderate-to-high positive psychological change, the temporal order between coping strategies, deliberate rumination, family support, and positive psychological change could not be determined. Therefore, these factors should be interpreted as correlates of positive psychological change rather than causal predictors. Future longitudinal studies are needed to examine how positive psychological change changes over time and how changes in psychosocial resources and cognitive processing are temporally related to changes in positive psychological change.

Second, the study relied on self-reported questionnaires and interviews, which may have introduced recall bias, social desirability bias, and common method bias. Participants may have underreported negative experiences or overreported positive psychological changes, especially because positive psychological change involves personal reflection on sensitive and stressful experiences. Although validated instruments and qualitative interviews were used to collect data from different angles, positive psychological change should not be implied as direct evidence of objective psychological improvement.

Third, this study did not include a comprehensive measure of COVID-19-related trauma exposure or perceived threat. Although selected COVID-19-related experiences were assessed, including personal infection, work-related quarantine, and loss of acquaintances or family members due to COVID-19, the survey did not collect detailed information on the intensity, duration, and subjective impact of pandemic-related exposure. For example, direct contact with infected patients, workload intensity during specific outbreak periods, fear of infection, separation from family members, detailed bereavement experiences, and perceived threat during work were not assessed. Therefore, the findings should be interpreted as positive psychological change reported in the post-pandemic occupational context of CHWs, rather than as evidence that all participants experienced the COVID-19 pandemic as a traumatic event in the same way.

Fourth, this study focused on positive psychological change and did not measure posttraumatic depreciation, traumatic stress, PTSD symptoms, or other negative psychological outcomes, such as burnout, depression, or anxiety. Positive psychological change can coexist with psychological distress, and positive psychological change does not necessarily indicate the absence of adverse mental health effects. Because traumatic stress and PTSD symptoms were not assessed, this study could not determine whether all participants experienced the COVID-19 pandemic as a traumatic event, nor could it distinguish whether the reported positive changes reflected positive psychological change following traumatic stress, normal adaptation to a prolonged stressful work period, or both. Consequently, this study could not provide a complete picture of both positive and negative psychological responses among CHWs after the COVID-19 pandemic. Future studies should assess positive psychological change together with trauma exposure, perceived threat, PTSD, posttraumatic depreciation, and other mental health outcomes to better understand the psychological consequences of public health emergencies.

Fifth, the measurement of positive psychological change has several limitations. This study used the 20-item C-PTGI rather than the more recently developed PTGI-X. Although the C-PTGI has been widely used in Chinese studies and demonstrated high internal consistency in the present sample, the removal of the religious faith item may have limited the assessment of spiritual-existential aspects of positive psychological change and reduced comparability with studies using the PTGI-X. In addition, we did not perform a full psychometric evaluation of the C-PTGI among Chinese CHWs, such as confirmatory factor analysis or measurement invariance testing. Furthermore, moderate-to-high positive psychological change was defined using an operational cutoff based on the PTGI response scale rather than a validated clinical threshold. Although sensitivity analyses using the continuous score yielded generally consistent findings for the major psychosocial factors, the cutoff-based classification may have influenced prevalence estimates and the interpretation of logistic regression results. Future studies should further validate PTGI-X or other culturally appropriate positive psychological change measures among Chinese primary care workers and examine whether different scoring approaches produce consistent findings.

Sixth, although the qualitative phase provided detailed explanations of CHWs’ perceived psychological changes, the qualitative sample included 12 participants from 6 CHCs in Shenzhen. This sample size is consistent with the idiographic focus of IPA, but it may not capture all possible experiences among CHWs with different professional roles, institutional conditions, or pandemic-related work exposures. In addition, participants who were more willing to discuss their psychological experiences may have been more likely to take part in the interviews.

Finally, the study was conducted in Shenzhen, a large and economically developed city in southern China, and focused on active healthcare technical staff working in CHCs. Therefore, the findings may not be generalizable to other CHC-affiliated staff, CHWs in less developed regions of China, or primary care workers in other countries. Although the quantitative phase used cluster sampling across districts and the qualitative phase included participants with diverse professional backgrounds, we did not collect detailed center-level information, such as service volume, catchment population, patient characteristics, or the number of respondents from each CHC. As a result, we could not calculate CHC-specific response rates, compare respondents and nonrespondents at the CHC level, or formally evaluate center-level heterogeneity and clustering effects. In addition, although included and excluded respondents showed similar baseline characteristics, the exclusion of 355 returned questionnaires (35.9%) may still introduce potential selection bias related to unmeasured characteristics. These factors may limit the representativeness of the sample and the generalizability of the findings. Future studies in more diverse primary care settings are warranted to determine whether similar patterns of positive psychological change and associated factors are observed in other populations.

## 5 Conclusion

This mixed-methods study found that more than half of CHWs in Shenzhen reported moderate-to-high positive psychological change in the post-pandemic period. Positive coping, deliberate rumination, and family support were significantly associated with moderate-to-high positive psychological change. The qualitative findings further showed that CHWs described positive psychological changes in personal strength, interpersonal relationships, appreciation of life, new possibilities, and reflections on life and values, and helped explain how coping strategies, cognitive processing, and family support were related to these changes. These findings suggest that positive psychological change among CHWs after the COVID-19 pandemic should be understood in relation to both individual psychological processes and the social context in which CHWs worked and lived. Future workplace mental health programs for CHWs should support coping skills, reflective processing of difficult work experiences, and family-sensitive support. Strengthening these resources may help protect CHWs’ mental health and improve the preparedness of primary care systems for future public health emergencies.

## Data Availability

The datasets presented in this article are not readily available because the data cannot be shared publicly due to concerns about participant confidentiality and privacy. De-identified individual-level data are available for researchers who meet the criteria for access to confidential data and upon approval of a data use proposal. Requests to access the datasets should be directed to Qingyu Li, liqy24@mails.tsinghua.edu.cn.
