# Supplementary material for: Positive psychological changes and associated factors among community health workers post-COVID-19 in China: a mixed-methods study
Source: Front Public Health. 2026 Aug 28;14:1918732. doi: 10.3389/fpubh.2026.1918732 (PMC13561946; doi:10.3389/fpubh.2026.1918732)
Supplement: Supplementary file 1 [file Table_1.DOCX]

**Supplement 1**

**eTable 1** Geographic distribution and eligible CHW numbers of the 51 selected CHCs

**eTable 2** Comparison of baseline characteristics between included and excluded participants

**eTable 3** Interview guideline for qualitative research phase

**eTable 4** Univariate and multivariate linear regression on PTG and selected variables

**eTable 5** Distributions of background variables in qualitative phase

**eTable 6** Themes, subthemes, and representative quotations of factors associated with PTG in the qualitative phase

**eTable 1 Geographic distribution and eligible CHW numbers of the 51 selected CHCs**

| **Administrative district** | **Number of selected CHCs** | **Number of eligible CHWs invited** | **Percentage of invited CHWs, %** | **Median eligible CHWs per CHC** | **Range** |
| --- | --- | --- | --- | --- | --- |
| **Luohu** | 7 | 211 | 16.1 | 22.0 | 18–69 |
| **Futian** | 12 | 287 | 21.9 | 20.5 | 14–52 |
| **Nanshan** | 3 | 78 | 5.9 | 25.0 | 21–32 |
| **Bao’an** | 10 | 227 | 17.3 | 18.5 | 13–37 |
| **Longgang** | 8 | 201 | 15.3 | 23.5 | 13–43 |
| **Yantian** | 1 | 20 | 1.5 | 20.0 | 20–20 |
| **Longhua** | 4 | 163 | 12.4 | 39.5 | 34–50 |
| **Pingshan** | 2 | 27 | 2.1 | 13.5 | 9–18 |
| **Guangming** | 3 | 75 | 5.7 | 26.0 | 19–30 |
| **Dapeng** | 1 | 22 | 1.7 | 22.0 | 22–22 |
| **Total** | 51 | 1,311 | 100.0 | 22.0 | 9–69 |

**eTable 2 Comparison of baseline characteristics between included and excluded participants**

| **Variables** | **Eligible (N=634)** | **Ineligible (N=355)** | **p-value** |
| --- | --- | --- | --- |
| **Age** | 35.7±8.2 | 36.1±8.2 | 0.175 |
| 18-25 | 47(7.4) | 30 (8.5%) |  |
| 26-35 | 305(48.1) | 122 (34.4%) |  |
| 36-45 | 190(30.0) | 148 (41.7%) |  |
| 46-55 | 83(13.1) | 50 (14.1%) |  |
| >55 | 9(1.4) | 5 (1.4%) |  |
| **Gender** |  |  | 0.851 |
| Male | 152(24.0) | 87 (24.5%) |  |
| Female | 482(76.0) | 268 (75.5%) |  |
| **Marital status** |  |  | 0.582 |
| Married/living with partner | 444(70.0) | 255 (71.8%) |  |
| Unmarried/divorced/separated/widowed | 190(30.0) | 100 (28.2%) |  |
| **Monthly income (CNY)** ^a^ |  |  | 0.223 |
| <5,000 | 29(4.6) | 8 (2.3%) |  |
| 5,001-8,000 | 109(17.2) | 51 (14.4%) |  |
| 8,001-10,000 | 159(25.2) | 103 (29.0%) |  |
| 10,001-15,000 | 208(32.8) | 123 (34.6%) |  |
| 15,001-20,000 | 80(12.6) | 48 (13.5%) |  |
| >20,000 | 49(7.7) | 22 (6.2%) |  |
| **Education level** |  |  | 0.761 |
| Technical secondary school or junior college | 64(10.1) | 35 (9.9%) |  |
| Undergraduate | 508(80.1) | 280 (78.8%) |  |
| Master degree and above | 62(9.8) | 40 (11.3%) |  |
| **Occupation** |  |  | 0.256 |
| Doctor | 308(48.6) | 188 (53.0%) |  |
| Nurse | 228(35.9) | 124 (34.9%) |  |
| Others ^b^ | 98(15.5) | 43 (12.1%) |  |
| **Professional title** ^c^ |  |  | 0.133 |
| Primary title | 218(34.4) | 100 (28.2%) |  |
| Junior title | 358(56.5) | 219 (61.7%) |  |
| Senior title | 58(9.1) | 36 (10.1%) |  |

^a^ CNY: Chinese yuan, 1USD≈7CNY.

^b^ Others include pharmacists, imaging technicians, and other healthcare technical personnel.

^c^ Primary titles include community health workers at both the primary titles and those without specific titles. Senior titles encompass community health workers holding associate senior titles as well as those with senior titles.

**eTable 3 Interview guideline for qualitative research phase**

| **Topic domain** | **Example question** |
| --- | --- |
| **Introduction to interview objective** | - This study aims to understand your psychosocial changes experienced after experiencing the COVID-19 pandemic, and explore possible improvement measures to enhance the physical and mental health of community health workers…Your personal information will be kept confidential; we will not disseminate or disclose any of your personal information. It will be restricted to use by the research team only and will be destroyed promptly after the conclusion of the study…If you have no further questions after reading this, please sign at the end of the informed consent form. |
| **Basic information** | - What is your date of birth? - Are you married? Do you have children? |
| **Work-related information** | - What are the main responsibilities of your current position? - Have your responsibilities changed since the COVID-19 outbreak? |
| **Changes after** **experiencing the COVID-19 pandemic** | - Prior to COVID-19, had your thinking about the meaning of life changed? - Prior to COVID-19, have there been any changes in your interpersonal relationships? |
| **Potential factors** **associated with PTG** | - Do you often think about work and life over the past three years? - How do you cope with these difficulties and pressures? - Have you received support from others or your organization? |

**eTable 4.** **Univariate and multivariate linear regression on positive psychological changes and selected variables (N = 634)**

| **Independent variables** | **positive psychological changes score** | | | |
| --- | --- | --- | --- | --- |
|  | **Univariate b (95%CI)** | ***P* (Uni)** | **Multivariable b (95%CI)** | ***P* (Multi)** |
| ***Social demographic variables*** |  |  |  |  |
| Age (years) | 0.014 (-0.165, 0.193) | 0.881 | -0.072 (-0.264, 0.120) | 0.461 |
| Gender (female versus male) | 0.961 (-2.460, 4.383) | 0.581 | -0.532 (-3.845, 2.782) | 0.753 |
| Marital status (married versus unmarried) | 0.940 (-2.248, 4.129) | 0.563 | -1.070 (-4.231, 2.090) | 0.506 |
| Education level (master or above versus college or below) | -0.820 (-5.739, 4.098) | 0.743 | -0.429 (-4.951, 4.092) | 0.852 |
| Monthly income (>10000 versus ≤10000 CNY) | -0.398 (-3.326, 2.530) | 0.789 | -0.913 (-3.566, 1.740) | 0.499 |
| Occupational type (reference: others) |  |  |  |  |
| Nurse | -3.761 (-8.196, 0.674) | 0.096 | -0.888 (-4.776, 3.000) | 0.654 |
| Doctor | -3.955 (-8.213, 0.304) | 0.069 | -2.230 (-6.150, 1.690) | 0.264 |
| Technical qualification  (junior title and above versus primary title) | -1.178 (-4.253, 1.897) | 0.452 | -0.980 (-4.233, 2.273) | 0.554 |
| ***Variables related to COVID-19*** |  |  |  |  |
| Families died due to COVID-19  (Yes versus No) | 0.766 (-2.704, 4.236) | 0.665 | -2.389 (-5.325, 0.548) | 0.111 |
| Quarantine experience due to COVID-19  (Yes versus No) | **7.215 (4.095, 10.335)** | **<0.001** | **4.146 (1.477, 6.814)** | **0.002** |
| COVID-19 infection | **3.837 (0.143, 7.531)** | **0.042** | 2.053 (-1.089, 5.195) | 0.200 |
| ***Psycho-social variables*** |  |  |  |  |
| Negative coping (score) | -0.266 (-0.478, -0.055) | 0.014 | 0.012 (-0.193, 0.218) | 0.906 |
| Positive coping(score) | **1.510 (1.283, 1.736)** | **<0.001** | **1.116 (0.873, 1.360)** | **<0.001** |
| Intrusive rumination (score) | **0.287 (0.015, 0.560)** | **0.039** | -0.193 (-0.534, 0.149) | 0.269 |
| Deliberate rumination (score) | **0.863 (0.604, 1.123)** | **<0.001** | **0.952 (0.628, 1.276)** | **<0.001** |
| Family support (score) | **1.427 (1.147, 1.706)** | **<0.001** | **0.675 (0.310, 1.041)** | **<0.001** |
| Friend support (score) | **1.684 (1.372, 1.995)** | **<0.001** | **0.482 (0.019, 0.945)** | **0.041** |
| Others support (score) | **1.439 (1.136, 1.742)** | **<0.001** | -0.067 (-0.500, 0.366) | 0.761 |

**eTable 5 Distributions of background variables in qualitative phase**

| **No.** | **Gender** | **Age** | **Occupation** | **Marital status** | **Child** | **Educational level** |
| --- | --- | --- | --- | --- | --- | --- |
| A | Female | 42 | Nurse | Married | Yes | Undergraduate |
| B | Female | 33 | Doctor | Married | Yes | Undergraduate |
| C | Female | 39 | Doctor | Married | Yes | Undergraduate |
| D | Female | 49 | Nurse | Married | Yes | Undergraduate |
| E | Male | 30 | Doctor | Unmarried | No | Undergraduate |
| F | Male | 28 | Doctor | Unmarried | No | Undergraduate |
| G | Female | 39 | Doctor | Married | Yes | Undergraduate |
| H | Female | 50 | Nurse | Married | Yes | Undergraduate |
| I | Male | 27 | Other CHW*^1^* | Unmarried | No | Undergraduate |
| J | Female | 30 | Other CHW*^1^* | Married | Yes | Junior college |
| K | Male | 30 | Doctor | Married | No | Master |
| L | Female | 27 | Nurse | Unmarried | No | Undergraduate |

CHW: community health worker

*^1^*Other CHW: pharmacists, imaging technicians, and other healthcare technical staff

**eTable 6** **Themes, subthemes, and representative quotations of factors associated with positive psychological changes in qualitative phase**

| **Theme** | **Subtheme** | **Sentence** |
| --- | --- | --- |
| **Personal Resources** | Personality Traits | *"I think I am (optimistic and positive) because I often tell my subordinates that no matter how difficult things are, being afraid is useless. When it comes, you have to face it head-on (A, female, nurse)."* |
|  | Unique Experiences | *"Our generation, being mostly only children, tends to be quite independent in work and life. But the pandemic, calling together people who might not know each other, just like how we supported each other during the pandemic, with different units coming together, made me realize that unity truly makes a difference (C, female, doctor)."* |
|  |  | *"The old lady, she was very irritable and had a bad temper when she first came... But after talking to her more, she started to listen to me. I would chat with her about hypertension and other professional issues during my rounds. When she left, she even left us a thank you letter(E, male, doctor)."* |
| **Social support** | Unit support | *"For instance, when I was newly pregnant, which was quite an unstable period with some bleeding in the early stages, the organization actually provided additional support. I wasn't placed in the first group, allowing me to stabilize gradually (B, female, doctor)."* |
|  | Family support | *"My parents-in-law help with the kids. If it were just me, I couldn't manage it. My husband also supports my work; he doesn't complain about my overtime or that I neglect the children, which is quite manageable (J, female, other CHW)."* |
|  |  | *"My family is quite supportive because they understand the nature of my work and the unique demands of these times. Fortunately, as I only have one child, both my parents and my in-laws help out. They take care of my child, so I don't have to worry about home issues, which really alleviates a lot of my concerns(C, female, doctor)."* |
| **Coping Strategies** | Negative coping | *"You can only do this, you have no choice but to do it (L, female, nurse)."* |
|  | Proactive Actions | *"I quite like philosophy, and last year, I revisited some classical works of the Confucianism, Buddhism, and Taoism, and learned about Wang Yangming's philosophy of the mind. After organizing these philosophical ideas, I was able to see things in a new light(F, male, doctor)."* |
|  |  | *"No matter how difficult things are, being afraid is useless. When it comes, you have to face it... If the pressure is too much, just go out for a meal and do some shopping. For us, shopping can also alleviate some stress(A, female, nurse)."* |
| **Cognitive Processing** | Rumination | *"From the beginning to the end, we were fully involved. Of course, we went through many things, so some particularly unforgettable incidents sometimes come to mind, making me feel that it was a very special experience... Sometimes, when I think about a certain event, I realize how we were back then and how much better the situation is now (C, female, doctor)."* |
|  | Perception of Stress | *"During the pandemic, there was more mental stress... Sometimes we would suddenly receive an order, and we might need to conduct nucleic acid testing all night, which never happened before... Issues with personnel deployment, and we had to communicate more with the community, including the allocation of resources and dealing with the psychological conditions of the personnel (G, female, doctor)."* |
|  |  | *"At night, dealing with overwhelming tension, I've been at the station for a year. After coming back, I always have insomnia and can't sleep. Then, after a while of adjustment and taking sleeping pills, I really felt very tense at night(E, male, doctor)."* |
